# Pharmacogenomic Biomarkers of Follicle-Stimulating Hormone Receptor Malfunction in Females with Impaired Ovarian Response—A Genetic Survey

**DOI:** 10.3390/jcm10020170

**Published:** 2021-01-06

**Authors:** Alireza Tafazoli, Sławomir Wołczyński, Natalia Wawrusiewicz-Kurylonek, Seyed-Alireza Esmaeili, Wojciech Miltyk

**Affiliations:** 1 Department of Analysis and Bioanalysis of Medicines, Faculty of Pharmacy with the Division of Laboratory Medicine, Medical University of Białystok, 15-089 Białystok, Poland; Alireza.Tafazoli@umb.edu.pl; 2 Clinical Research Centre, Medical University of Białystok, 15-276 Bialystok, Poland; 3 Department of Reproduction and Gynecological Endocrinology, Medical University of Białystok, 15-222 Białystok, Poland; slawek.wolczynski@gmail.com; 4 Department of Clinical Genetics, Medical University of Białystok, 15-089 Białystok, Poland; natalia.kurylonek@gmail.com; 5 Immunology Department, Faculty of Medicine, Mashhad University of Medical Sciences, 9177948564 Mashhad, Iran; imunoman2009@gmail.com; 6 Immunology Research Center, Mashhad University of Medical Sciences, 9177948564 Mashhad, Iran

**Keywords:** FSHR, pharmacogenomics, personalized drug therapy

## Abstract

Follicle-stimulating hormone receptor (FSHR) plays an essential role as one of the most important molecules in response to some of infertility related medications. Impaired ovarian reserve and poor response to such treatments are partially dependent on the FSHR molecule itself. However, the function and drug sensitivity for this receptor may change due to various allele and polymorphisms in the *FSHR* gene. Studies indicated some of the FSHR-mediated treatments utilized in clinical centers display different outcomes in specific populations, which may arise from FSHR altered genotypes in certain patients. To support the increased demands for reaching the personalized drug and hormone therapy in clinics, focusing on actionable variants through Pharmacogenomic analysis of this receptor may be necessary. The current study tries to display a perspective view on genetic assessments for Pharmacogenomic profiling of the *FSHR* gene via providing a systematic and critical overview on the genetics of FSHR and its diverse responses to ligands for infertility treatment in females with impaired ovarian responses and show the potential effects of the patient genetic make-up on related binding substances efficacy. All identified functional drug-related alleles were selected through a comprehensive literature search and analyzed. Advanced technologies for the genetic evaluation of them are also discussed properly.

## 1. Introduction: FSHR, Related Infertility Medicines, and the Role of Pharmacogenetics

Follicle-stimulating hormone receptor (FSHR) is a rhodopsin-like receptor, which belongs to the G-protein-coupled receptors (GPCR) superfamily, and consists of an intracellular domain and a large leucine-rich repeat extracellular domain and passes seven times through the cellular membrane [1]. The *FSHR* gene consists of 10 exons, of which exon 1 to exon 9 encode the extracellular domain and exon 10 is responsible for a small part of the extracellular domain and transmembrane and intracellular parts. FSHR binds the gonadotropin follicle-stimulating hormone (FSH) and other agonists leading to rapid activation of different cellular cascades, mainly cAMP–adenylyl cyclase–protein kinase A (also known as the adenylyl cyclase), Ras-MAPK (mitogen-activated protein kinase), and β-arrestin signal transduction pathways. Adenylyl cyclase and RAS-MAPK are activated through activation of heterotrimeric Gs protein and adaptor proteins, recruiting adenylyl cyclase, phospholipase C, and guanine nucleotide exchange factors (GEFs) respectively [2,3]. In addition, other related transcription factors, as well as cAMP regulatory element-binding protein (CREB) and extracellular signal-regulated kinase (ERK), cause the final fertility effects of activated FSHR such as maturation and follicular development in addition to the differentiation of ovaries by targeting the promoter of some genes and inducing up/downregulation of them (i.e., *CYP19A1*) [4].

It has been estimated that globally infertility affects between 8–12% of couples of their reproductive age (over 186 million people). However, males and females contribute differently to overall infertility cases [5]. Many FSHR associated treatments like synthetic or natural derived follicle-stimulating medicines, gonadotropins, and ovarian stimulation drugs such as clomiphene and metformin are used for infertility treatment in females [6]. These drugs are also used in assisted reproductive technologies (ART) to stimulate multiple follicles, which need the medications to collect, to develop, and to maturate eggs with different dosages and usage protocols [7]. The differences are because of the various responders in women who received these treatments during infertility therapy approaches. In addition, molecular biology of the triggered cellular pathways for these medications turned to more attention in clinical investigations. From this point of view, FSH and other related medicines as the specific ligands bind to FSHR, leading to start the signal transduction pathways in target cells (granulosa cells in females). This leads to the growth of follicles in ovaries and final ovulation through changes in expression of particular genes and thus triggering proliferation and differentiation of oocyte in females. Studies on side effects, tolerance, drug safety and efficacy, and treatment preferences by different physicians alongside the demographic characters of patients indicated poor to an increased response to FSHR coupled medicines and sometimes even life-threatening excessive response in some patients [8,9,10]. Indeed, the relationship between infertility treatments and some types of cancers due to the different drug safety and toxicity is still controversial and needs further investigation and long-term follow-up studies [11]. Poor responders or women with impaired ovarian reserve, however, are those patients who display at least two of Bologna three criteria: a) Advanced maternal age, (b) Previous poor ovarian response after ovarian stimulations, and (c) Abnormal ovarian reserve tests. Women with the age of 40 and retrieval of oocytes ≤ three are considered as the cutoff values for discrimination of poor ovarian responders. The European Society of Human Reproduction and Embryology (ESHRE) also included the antral follicle count (AFC) and anti-Müllerian hormone (AMH) tests result with the variable levels of <5–7 follicles or <0.5–1.1 ng/mL, correspondingly [12]. Other developed classifications of infertility patients focused on some specific features as well as women’s age and provided a more nuanced picture of poor ovarian response as a guide for physicians in patient management. The latter is named as the patient-oriented strategies encompassing individualized oocyte number criteria (POSEIDON) and used for diagnosis and management of low prognosis patients who received ART in the clinic [13,14]. The above-mentioned criteria help the clinicians to categorized different responders with miscellaneous ovarian stimulation outcomes. Such variability also appeared through FSH and other hormonal therapies in infertility centers. The diverse response of patients to recombinant/urinary ligand as FSH plus other FSHR related treatments also have been reported in different regions (some examples are listed in Section 3).

Pharmacogenetics and genomics (PGx) studies also serve as the main path for achieving the best medication(s) for patients with maximum safety and efficacy. Various methods have been employed to identify the genetic landscape related to pharmacogenes, which are responsible for the metabolism of certain drugs and their targets in the body, lead to different prescription adjustments in patients for finding the optimal dosage. With the emergence of high-throughput sequencing technologies, finding the potential genetic variation(s) that influence drug responses in patients would be fast and cost-effective. The ultimate goal for such evaluations in patients is reflected by the famous quote “choosing the right drug with the right dosage for the right person at the right time”, as it is mentioned for a while in the personalized medicine considerations [15,16]. To support the increased demands for reaching the personalized drug and hormone therapy in infertility clinics, focusing on actionable/potentially actionable variants through PGx analysis of FSH receptor may be necessary. This review provides a critical and systematic analysis of the genetic basis for FSHR and the functional alterations due to various alleles in the genome, resulting in diverse responses to associated ligands during infertility treatment in females with impaired ovarian reserve because of poor response to FSH and FSHR related stimulators. In addition, the related cellular pathway genes are listed, and the potential effects of them on related drugs’ efficacy are discussed. The latest identified functional alleles for the *FSHR* gene are analyzed, and advanced technologies for genetic evaluation of them are introduced as well.

## 2. Methods

**Figure 1:**
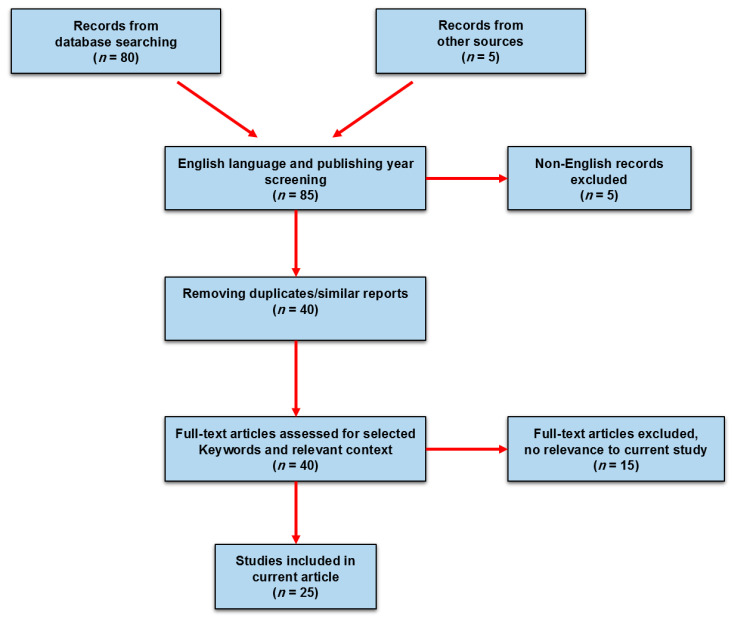
Literature search and inclusion/exclusion criteria for systematic reporting of pharmacogenetics and genomics (PGx) studies in follicle-stimulating hormone receptor (FSHR) malfunction in females with poor ovarian response.

A literature search was conducted in bibliographic databases (PubMed, Web of Science and Scopus) and gray literature using keywords: “*infertility and personalized medicine”*, “*FSHR drugs”*, “*FSHR pharmacogenetics”*, “*FSHR polymorphisms and mutations”*, and “*pharmacogenetics of infertility”* for studies published after 2000, in order to provide an exhaustive report for all the introduced functional variants so far. Among the 85 yielded results, after removing duplicate/similar reports and rolling out non-English language articles, 40 articles were included for further assessments. In the next step, first, the abstracts were screened if the selected keyword expansion were related to our study context, followed by full-text article assessment for those papers of direct implication on FSHR genetic landscape and PGx analysis of infertility-related drugs for females. Ultimately 25 papers were marked, quality assessment was done, and risk of bias was considered based on PRISMA guideline for constructing this review (Figure 1).

## 3. A Quick Overview on Pharmacogenomics of *FSHR* in Females with Poor Ovarian Response

**Figure 2:**
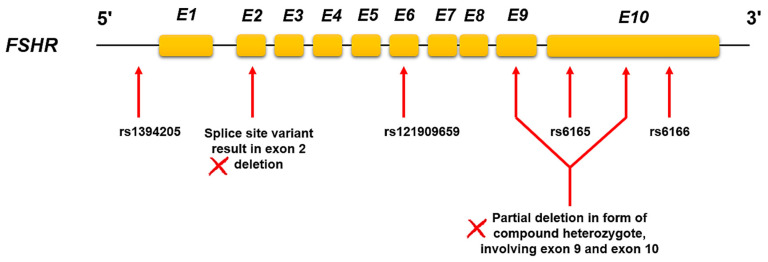
All the actionable and/or functional validated PGx variants in the *FSHR* gene. Pharmacovariants introduced here are based on clinical reports for changing the ovarian responses observed during infertility treatment procedures in females in different populations *(see the text and* Table 1 *for more details)*. FSHR, follicle-stimulating hormone receptor; PGx, pharmacogenomics.

Through 40 fully scrutinized publications in this review, 25 of them showed the relevant impact of *FSHR* genotype on infertility treatment outcomes in the clinic. While they are introducing the actionable genetic alterations (mutations and polymorphisms) in the *FSHR* gene with direct effects on medication applying in females with poor ovarian responses, the dosage adjustment and considerations may be purposed after the genetic testing in such patients. Here, we first have an overview of the different genetic profiles of *FSHR* and their clinical evidence-based, actionable PGx properties in different populations (Figure 2 and Table 1). These are validated genetic variants in the *FSHR* gene, which may need treatment modifications in patients, provided in the form of some examples for such studies. The complete explanation on PGx of *FSHR* and related pharmacovariants are provided in Section 4. In addition, we list some of the diverse responses to FSHR connected treatments in different populations in a detailed Table 2 as examples of such differences in FSHR function because of these genetic profiles in patients. However, due to the lack of similar association between the variants and the drugs in different studies, in which treatment modifications implemented according to the specific type of variants in the *FSHR* gene and also for avoiding any incompatibility through the issue, some information, as well as applied FSH dosages, duration of applying, and so on, are not reported in the current paper. Next, we talk about the latest genetic test methods for finding genetic variations of *FSHR* (next-generation sequencing technologies and advanced single nucleotide polymorphism (SNP)-array analysis). Finally, we provide an overview of the cellular pathway genes, which may show interaction and potential impact on selected drugs’ efficacy.

## 4. Genetic and Pharmacogenetic Variations in *FSHR* Gene

Different inactivating or hyper-activating genetic alterations such as loss or gain of function mutations and polymorphisms were displayed in coding, regulatory, and splice sites of the *FSHR* gene, leading to receptor malfunction in the form of reduced cell surface expression level, ligand-binding, and total function in target cells with the relative impact on fertility issues in females. This also is part of the reasons for disorders like premature ovarian failure, polycystic ovary syndrome (PCOS), ovarian hyper-stimulation syndrome (OHSS), and also diverse drug responses in some patients [35,36,37]. Specifically, there are several cases of OHSS due to gain of function mutations of the *FSHR* gene. Activating mutations resulted in a predisposition to OHSS, suggesting that ovarian response may depend on the *FSHR* genotype too. In addition, studies have demonstrated the p.680Asn/Ser polymorphism of the FSHR could be considered as a predictor for the severity of symptoms in patients who develop OHSS [38]. However, the distribution of related alleles is significantly different in various ethnic groups and populations [29,39]. The gain of function mutations was seen mostly in the extracellular domain as an autosomal dominant variant, while the loss of function alterations affects all parts of the receptor in extra- and intracellular in addition to transmembrane sections, mostly as an autosomal recessive or dominant-negative in some cases [40]. While the mutations are rare and the clinical outcomes for them would be expected or predicted easier, the *FSHR* gene polymorphisms as the more common and/or prone to ignore variants in patients with impaired ovarian response were investigated thoroughly for possible impact on reproductive ability. Such studies indicated both types of variations are distributed in different parts of the *FSHR* gene and can cause various effects and outcomes in patients. For example, a splice site variant, which results in exon two deletions in the *FSHR* gene and affects the extracellular domain of FSHR protein and the reduced response of the receptor, was found in women who received infertility treatment. In vitro analysis of this particular variant displayed the formation of functional heterodimers with the wild-type receptor when co-expressed alongside it, which lead to the reduced activity for normal receptor [22,23]. In addition, mutations in the leucine-rich region of the FSHR extra domain result in the reduction of agonist-binding, affecting the drug responses in patients [41,42].

Generally, the mutations and polymorphisms in *FSHR* genes bring about diverse activity of this receptor for infertility medicines and treatment procedures as well as IVF and ICSI (intra-cytoplasmic sperm injection) in females. Two c.919G>A (p.307Thr/Ala) and c.2038G>A (p.680Asn/Ser) single nucleotide variants (SNVs) indicated as well-known functional variants in the *FSHR* gene coding region with different frequencies in populations, showed the associations with FSH and FSH derived infertility drugs response for ovarian stimulation during infertility treatment. Linkage disequilibrium between these two alleles will help to determine the presence of one according to another in individuals. In addition, the association of these two alleles as a distinct diplotype with the ovarian response to FSH stimulation in women who undergo assisted reproduction procedures explored, and the result indicated the significant relationship between the diplotype and ovarian response too [43]. Other characterized functional variants for females’ diverse response to ovarian stimulation drugs include g.-29G>A in the 5′ UTR of the *FSHR* gene, which affects the level of gene expression and influences ligand-binding measures in patients [20,31]. These alleles are considered the variations with PGx effects in the *FSHR* gene during infertility treatment approaches, and the consistent data for retrieved oocytes, stimulation duration, FSH consumption, etc., are provided for them adequately. For example, AA homozygous genotype for rs6165 (c.919A>G) indicated more retrieved oocytes and shorter stimulation time compared to the other two genotypes, the AG heterozygotes and GG homozygous in patients who underwent controlled ovarian stimulation protocols. In addition, GG homozygous and AG heterozygotes of *FSHR* rs1394205 (g.-29G>A) showed a significantly lower amount of FSH consumption during the ART procedures [21]. The GG homozygous form for rs6166 (c.2038G>A) showed a higher level of FSH than AA homozygous and AG heterozygotes in PCOS patients [44]. However, such patients are more prone to hyperstimulation during the treatments, instead of a poor ovarian response. Other studies, however, reported the GG homozygous of rs6166 would also be associated with poor response to exogenous FSH and related drugs and the need for a higher amount of them in order to show a good response [17].

Nevertheless, the number of *FSHR* genetic polymorphisms and variations is too much, and not all of them have been investigated or show such function in the female reproduction system [45]. For example, identified mutations with decreased or completely abolished FSH-binding effects in women with primary or secondary amenorrhea are including c.566C>T in exon 7 (p.189Ala/Val), c.1043C>G in exon 10 (p.348Pro/Arg), c.1222G>T in exon 10 (p.408Asp/Tyr), and c.671A>T in exon 7 (p.224Asp/Val) and are found in the extracellular domain, except c.1222G>T which is happening in the transmembrane domain of FSHR protein. Also, they categorized as inactivating mutations in the *FSHR* gene in women and validated through in vitro functional studies [46]. Yet, there is no/not enough clinical evidence for prescription modifications during the infertility treatment of the related patients based on these mutations. Furthermore, a recently identified *FSHR* novel variant (c.1268T>C (p.423Ile/Thr)) in the second transmembrane domain of the protein in a woman with primary ovarian failure was extensively investigated for receptor malfunction and any therapy dosage modifications. Applying in-vitro and in-silico approaches demonstrated the membrane expression level of FSHR was impaired, and the cAMP/PKA signaling pathway would be severely affected while β-arrestin-dependent ERK1/2 phosphorylation received less amount of impact. In conclusion, it has been shown that the mutation will cause FSHR dose-dependent cell signaling functional alterations and attenuated response [47]. Some loss of function mutations are also introduced before, but due to not determined FSH-binding and cell surface expression level of receptor, there was no report for the possible effects or interruption with drug intake and efficacy in patients (i.e., c.662A>T (p.221Val/Gly), c.1253T>G (p.418Ile/Ser), c.1298C>A (p.433Ala/Asp), c.1723C>T (p.575Ala/Val), etc.) [46].

## 5. Advanced Genetic Screening Methods for FSHR Profiling

Most of the genetic variations for the *FSHR* gene are defined as SNVs, and because of that, many of them could be included in genome-wide array-based genotyping (Infinium BeadChips from Illumina) and/or specified custom SNP array chips as part of infertility genetic profiling for the related patients (Affymetrix Axiom array) [48]. However, such methods are mostly performed just for male infertility cases [49]. Nowadays, traditional orthogonal genetic tests like real-time PCR or multiplex ligation-dependent probe amplification (MLPA) assay replaced by advanced high-throughput sequencing methods in clinical infertility centers. The utilization of such platforms resulted in faster and more accurate genetic screening and identification of possible underlying genetic reasons for FSHR malfunction in infertile patients for a lower price. As the next generation sequencing (NGS) test outcomes proved to be reliable with regards to both clinical validity and utility, the clinical applications for that would be more common in the near future. Today, NGS approaches and mostly targeted sequencing panels are employed by the clinicians and several genetic testing companies for genotyping the known group of infertility related genes or specific variants (including *FSHR*) in order to identify genetic signatures in infertile women [50,51,52]. These methods have been used successfully for the determination of *FSHR* SNVs and/or other genetic alterations like CNVs in addition to risk assessment in patients. In fact, NGS methods were recently utilized positively in *FSHR* profiling and novel variant identification in numerous studies [46,53,54,55]. Some examples also have been illustrated in the development of infertility NGS panel for targeted exons and their flanking regions in 75 infertility related genes, including *FSHR* as one of the diagnostic genes which have proven associations with infertility, in order to assess the genetic variations of infertile patients with a custom bioinformatic pipeline for data analysis. By applying the MiSeq platform of Illumina, investigators revealed the underlying genetic cause of infertility in their 25 samples properly [56]. França et al. also reviewed the ability of NGS methods in the discovery of heterogeneity of some FSHR and other infertility gene-related disorders and concluded the widespread usage of such technologies in the near future for the detection of new players in female reproduction diseases would be recommended [57]. While such results are truly satisfying, but many rare pharmacogenetic variants are heavily population-specific and ethnic background-dependent; more comprehensive sequencing technologies like whole exome and whole genome sequencing (WES and WGS) have also been proposed for profiling, decoding, and revealing any novel genetic variations in *FSHR* and other PGx related genes [58,59]. These techniques will be mostly useful for infertile cases with non-identified genetic variations in selected genes and also without other common encountered reasons in clinics. Here, the huge obtained data and performing correct genotype-phenotype correlations plus labor in vitro validation studies would be considered as the bottleneck for assigning them as the routine approaches in infertility centers. However, the specific bioinformatics and computational prediction tools have been introduced for such PGx data analysis and interpretation [60].

## 6. Genetic Variations in FSHR Related Cell Signaling Genes as Potential Players for Diverse Infertility Drug Response

Glycosylation of Asn174 or Asn276 in the extracellular domain of FSHR as an important post-translational modification allows the correct folding and conformational changes of the protein, which are required for high-affinity binding of FSH and/or other related ligands. While the binding activity in non-glycosylated mutated receptors will be disrupted, two different forms of glycosylation of FSHR also result in different activities for it, too [61,62]. Some introduced mutations in the *FSHR* gene like c.175C>T, (p.59Arg/Thr), c.573A>T, (p.191Asp/Ile), c.1555C>A, (p.519Pro/Thr), c.1760C>A as a compound heterozygote, (p.587Pro/His), etc. affect the FSH-induced cAMP pathway and were seen in conditions like primary amenorrhea [37]. However, when the FSH hormone or FSHR related drugs and synthetic elements as hydrophilic components bind to the receptor, distinct cell signaling pathways as well as adenylyl cyclase, Ras-MAPK, and β-arrestin will be activated. The core involved enzymes/proteins for these intracellular pathways are Gs alpha subunit, adenylyl cyclase, protein kinase A, cAMP-specific phosphodiesterase, extracellular signal-regulated kinases, and arrestin beta. The related genes are also *GNAS1, ADCY, PKA, PDE4, MAPK1,* and *ARRB1,* respectively [63]. Alongside variants in the *FSHR* gene, mutations in any of the above-mentioned cell signaling core genes can also potentially affect the FSHR intracellular function and cause different responses to treatments in some patients too. Although such mutations may rarely occur in infertile women, they can induce an impaired signaling pathway in a highly complex process of FSHR inducing molecular trafficking [64]. While PGx markers in these genes are introduced for other phenotypes and diseases, so far, there is no infertility annotated variants reported in PGx databases (PharmGKB and CPIC) for the listed genes (pharmgkb.org, cpicpgx.org). However, the consideration of such alleles during *FSHR* genotyping in women with diverse responses to infertility drugs will not be useless. Although the PGx analysis of them is not investigated by the clinical researchers, the potential influences must be taken into account, especially when there is no pathogenic variant identified in the *FSHR* gene, the variants identified only in these genes, and other common possible reasons for the observed negative treatment have been ruled out. Yet, more evidence is required in order to put such variants as part of the new FSHR related modifiers in the field of pharmacogenetics and genomics. Moreover, some of the FSHR medicines like menotropins and choriogonadotropin-alfa also use other cellular receptors as well as LHCGR too. The drug’s efficacy assessments based on genetic profiling would be more complicated in such situations and need extra caution and effort. For example, compound heterozygote of allele C in LHCGR-291 and FSHR-29 displayed to result in altered rFSH dosage, and the total amount of mature oocytes in IVF treated Caucasian patients [65]. However, all together, the genetics of FSH responsiveness in infertility treatment can rest not only on potential alterations of the *FSHR* gene via mutations or polymorphisms but also on a host of other core genes for intracellular signaling pathways too. In addition, epigenetic modifications may be involved in the process as well. The latter may occur due to environmental factors or aging.

## 7. Discussion

**Figure 3:**
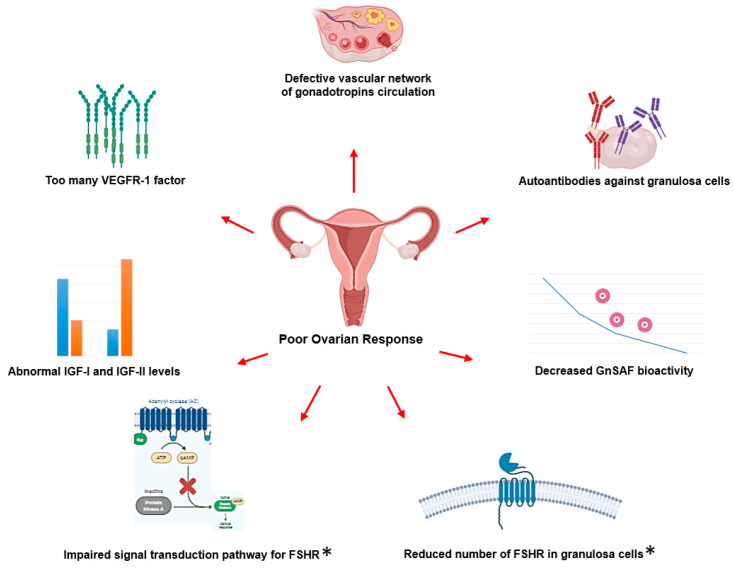
Suggested molecular mechanisms for poor ovarian response in females. The genetic landscape of individuals can potentially affect all of the introduced reasons here. Pharmacogenomic profiling for the reduced number of FSHR and impaired signal transduction pathway of that in patients before the implementation of ovarian stimulation proved to be beneficial in females undergoing infertility treatments. *, PGx implication. VEGFR-1: vascular endothelial growth factor receptor 1; IGF-I, II: insulin-like growth factor 1, 2; GnSAF: gonadotropin surge-attenuating factor

It has been reported that 9–24% of females would be introduced as poor responders after the implementation of ovarian stimulation protocols. Different molecular mechanisms have been suggested for reduced and poor ovarian response [66]. Through various reasons which introduced in different patients, a reduced number of FSHR molecules and impaired signal transduction pathway of that in some patients demonstrated to be directly relative to their genetic make-up (Figure 3).

Hence, despite common clinical assessments of AMH and AFC, as the routine tests for prediction of ovarian altered/poor response in females, PGx analysis of the *FSHR* gene is still considered as a matter of worth in special occasions like failure assisted reproductive techniques. As the efficacy of such treatments proved to be related to personalized ovarian stimulation protocols for each patient, PGx assays also may play an essential role in predict the ovarian response before performing stimulation by FSHR related ligands, lead to decreasing the number of ovarian stimulation attempts and the optimization of drug/hormone therapy management [67,68]. However, while the introduced alleles and haplotypes for the *FSHR* gene have been widely studied and well-characterized in different populations, there are some reports which indicated not all of these variants could be traced to specific ethnic groups. For instance, Ilgaz et al. showed there are no significant differences between infertile women and healthy controls for the Asn680Ser variant in the Turkish population, and the genotype landscape is consistent for both groups [36]. In addition, García-Jiménez and colleagues from Mexico indicated a non-significant association between 5′ UTR g.-29G>A and ovary response in IVF-treated women with controlled ovarian stimulation (COS) [69]. However, most of the investigations indicated lower effects of altered FSHR function on the overall rate of ovarian stimulation and also, the number of studies for ethnic differences in FSHR responses is not high. Still, reports like Turkish and Mexican studies display the necessity of personalized and/or population-specific genotyping approaches for *FSHR* and other infertility related genes through advanced unbiased and untargeted sequencing methods in particular regions (also see Table 1 for other examples) [48].

In addition, the PGx annotation is provided for a few of *FSHR* genetic variations based on clinical findings. Yet, there may not be any dosage guideline or FDA drug label for them, as there is a paucity of evidence. PGx guidelines are usually provided when there is more than one clinical report, which is needed for dosage modifications through different populations or ethnic groups. For *FSHR*-related pharmacovariants, also the number of evidence would be the main criteria, but the fact that *FSHR* gene variants show potential influence on drug response through a change in ligand and exogenous agonist sensitivity still make it necessary to follow the genotyping approaches in order to predict the diverse outcome of the treatment for some special case of infertile women. Moreover, based on major signal transduction activity, most of the core genes in cellular pathways for FSHR drugs, listed here, show the potentially actionable variants too. Hence, PGx annotation for them could be expected to be reached in the near future in personalized infertility treatment approaches. However, the correct decision on dosage specifying for the relevant patients must be considered through comprehensive genetic analysis of both *FSHR* gene and related intracellular pathway genes for related drugs in addition to bearing in mind other main influential factors on ovary reserve in infertile women. The combined analysis of different polymorphisms in *FSHR* at the same time can also be introduced as a valuable tool for investigating and predicting the efficacy of ovulation induction protocols, especially in the group of patients with failed attempts [70]. Moreover, some studies displayed in addition to SNVs, there could be other genetic variants like CNVs for the *FSHR* gene with a direct impact on protein function and drug resistance. The Database of Genomic Variants (DGV—v107) provides such structural variations (SVs) for the *FSHR* gene in detail. Kuechler and her team also reported a 163kb partial deletion in the form of the compound heterozygote, involving exons 9 and 10 of the *FSHR* gene. Conformational and functional studies revealed complete loss of function of FSHR protein and also FSH resistance with clinical manifestations, as the exon 10 encodes all the transmembrane part of the receptor and exon nine engages in making the extracellular domain [24]. Such scenarios cause a specific type of challenges, as most of the sequencing approaches are designed for SNV detection in *FSHR* and other related infertility genes in the clinical practice. However, the above-mentioned cases need the utilization of long-read sequencers like PacBio or Nanopore systems for more accurate genetic profiling of individuals and unraveling the CNV signatures in patients’ genes. PGx analysis of CNVs alongside the common SNVs have been implemented for other drug-related genes earlier [71]. The same could be considered for *FSHR* and related genes too. The overall considerations result in the precise genetic scanning of this important gene as an effective and actionable pharmacogene for its related infertility-mediated treatments.

## 8. Conclusions

While it is in the end, oocyte quality which is the most important parameter and that is no doubt affected by a plethora of factors and resulted in different treatment outcomes, but *FSHR* genotyping is still considered as a promising PGx approach for personalized infertility therapy in women, who receive the related medicines and displayed negative efficiency [72], especially, when there are no other common reasons for non-successful infertility therapy procedures. Various mutations and polymorphisms have been reported for this important pharmacogene with different frequencies in diverse populations. However, not all of them influence the drugs and treatment efficacy. Yet, to avoid any misdiagnosis and/or mismanagement of impaired ovarian response patients, genetic profiling of *FSHR* should be taken into account in clinical infertility centers too. In addition, the genetic alterations of genes in signal transduction pathways with regard to FSHR function in granulosa cells in females with ovarian failure may contribute to changing the drug response and effects. This is a new PGx area for infertility drugs and needs more investigation and clinical evidence in order to bring new insight into the field of personalized medicine.

## Figures and Tables

**Table 1:** *FSHR* gene variations with clinical evidence for pharmacogenetic effects in different populations.

*Gene*	*Variants with PGx Effects*	*Nucleotide Changes*	MAF* (%)	*Variation Effects*	*Reference*
*FSHR*	rs6166	c.2038 G > A	0.3–0.4	Allele G: poor response to infertility drugs	[17,18]
*FSHR*	rs6165	c.919 G > A	0.3–0.4	Allele G: poor response to ligands (decreased sensitivity)	[19]
*FSHR*	rs1394205	-29 G > A	0.2–0.3	Allele A: reduced gene expression level leads to a decreased level of the response to drugs	[20,21]
*FSHR*	-	exon2 del	NA*	Reduced response of the receptor for infertility treatments due to impaired receptor transferring to the cell membrane	[22,23]
*FSHR*	-	exon9 and 10 del	NA	Loss of function of receptor lead to FSH resistance	[24]
*FSHR*	rs121909659	c.479 A > G,T	0.000025	Partial loss of function and impaired cell surface expression of FSHR causes the reduced response in patients (Classified as pathogenic SNP)	[25,26]

MAF*: minor allele frequency, NA*: not available; FSHR, follicle-stimulating hormone receptor; SNP, advanced single nucleotide polymorphism.

**Table 2:** Examples of diverse responses in different populations to assisted reproductive technologies (ART) treatments due to alterations in patients’ *FSHR* genetic make-up.

*Country*	*Study Population*	*FSHR* *Evaluated SNP(s)*	*Different Responders to the Treatments* *Due to FSHR Altered Function*	*Conclusion*	*Reference*
Slovenia	60 women undergoing ovarian stimulation were selected	−29 G > A c.2038 G > A	Poor Responders: 28.3% Normal Responders: 43.4% High Responders: 28.3%	The GG genotype in rs1394205 is associated with poor ovarian response to COH ^1^, and the related patients may require higher doses of rFSH for ovulation induction.	[27]
Spain	170 women undergoing controlled ovarian stimulation included	c.2038 G > A	Poor responders: 58.4% Normal Responders: - ^2^ High responders: 27.7%	Discrete set of genes and polymorphisms, including rs6166 in the FSHR gene, may partially explain the poor response to FSH hormone during controlled ovarian stimulation treatments.	[28]
Greece	79 sub-fertile women and 46 normo-ovulatory women with diverse respond to IVF were included	c.2038 G > A	Poor responders: 28% Normal Responders: 36.8% High responders: -	Good (normal) responder group had a statistically significant Asn/Ser heterozygous variant (rs6166) with more follicles and oocytes in patients.	[29]
Ukraine	374 women, including ovary dysfunction patients and healthy individuals with different treatment responses, were selected	c.919 G > A c.2038 G > A	Poor responders: 10.42% Normal responders: 10.7% High responders: - (Study also included control groups: 51.6%)	Combined allelic distribution for rs6165 and rs6166 (Ala307-Ser680/Ala307-Ser680) genotype should have an impact on the delineation of stimulation protocols.	[30]
Germany	161 ovulatory women below the age of 40 years with different FSH stimulation requirements were included	c.919 G > A c.2038 G > A	Poor responders: - Normal Responders: - High responders: -	The Asn/Ser heterozygote genotype for rs6166 was significantly more common in infertile patients with diverse ovarian response. Ovarian response to FSH stimulation depends on the FSHR genotype.	[31]
Germany	93 women (homozygous for Asn/Asn or Ser/Ser) undergoing controlled ovarian hyperstimulation in IVF and ICSI	c.2038 G > A	Poor responders: 25.80% Normal Responders: 74.19% High responders: -	Lower FSH receptor sensitivity due to p.N680S sequence variation in FSHR (rs6166) resulted in lower estradiol levels following FSH stimulation, which cause the patients to need to receive higher FSH doses.	[32]
Sweden and China	Systematic review and meta-analysis on special FSHR variants and altered ovarian response in women undergoing IVF	c.2038 G > A	-	FSHR polymorphism Ser680Asn (rs6166), through the other pharmacogenomics variants, is the most optimal biomarker for implementing in routine clinical practice.	[33,34]
Armenia origin	Case report of a woman with secondary amenorrhea and very high plasma gonadotropin concentrations (especially FSH)	c.479 A > G,T	Poor responders: 100% Normal Responders: - High responders: -	rs121909659 causes partial loss of function, and impaired cell surface expression of FSHR resulted in reduced response in COH. The study reminds us of the population-specific assessments of FSHR.	[25]
United States	35 women undergoing in vitro fertilization included	exon2 del exon6 del exon9 del intron 8 insertion	Poor responders: 8.5% Normal Responders: 68.57%% High responders: 22.85%	FSHR splicing variants, seen in women with a normal menstrual cycle that show an abnormal response to FSH stimulation described. Exon 2 deletion was associated with low ovarian response.	[22]

^1^ COH: controlled ovarian hyperstimulation, ^2^ -: not determined/not applicable, IVF: In vitro fertilization. FSHR, follicle-stimulating hormone receptor; FSH, follicle-stimulating hormone; ICSI, intra-cytoplasmic sperm injection

## Data Availability

Not applicable.

## References

[1] Ulloa-Aguirre A., Zariñán T., Pasapera A.M., Casas-González P., Dias J.A. (2007). Multiple facets of follicle-stimulating hormone receptor function. Endocrine.

[2] Yan K., Gao L.-N., Cui Y.-L., Zhang Y., Zhou X. (2016). The cyclic AMP signaling pathway: Exploring targets for successful drug discovery (Review). Mol. Med. Rep..

[3] Thatcher J.D. (2010). The Ras-MAPK Signal Transduction Pathway. Sci. Signal..

[4] Conti M. (2002). Specificity of the cyclic adenosine 3′,5′-monophosphate signal in granulosa cell function. Biol. Reprod..

[5] Borght M.V., Wyns C. (2018). Fertility and infertility: Definition and epidemiology. Clin. Biochem..

[6] Lindsay T.J., Vitrikas K.R. (2015). Evaluation and treatment of infertility. Am. Fam. Physician.

[7] Hrometz S.L., Gates V. (2009). Review of available infertility treatments. Drugs Today.

[8] Derman S.G., Adashi E.Y. (1994). Adverse Effects of Fertility Drugs. Drug Saf..

[9] Samplaski M.K., Nangia A.K. (2015). Adverse effects of common medications on male fertility. Nat. Rev. Urol..

[10] Conforti A., Cariati F., Vallone R., Alviggi C., de Placido G. (2017). Individualization of treatment in controlled ovarian stimulation: Myth or reality. Biochim. Clin..

[11] Momenimovahed Z., Taheri S., Tiznobaik A., Salehiniya H. (2019). Do the Fertility Drugs Increase the Risk of Cancer? A Review Study. Front. Endocrinol..

[12] Ferraretti A.P., La Marca A., Fauser B.C.J.M., Tarlatzis B., Nargund G., Gianaroli L., on behalf of the ESHRE working group on Poor Ovarian Response Definition (2011). ESHRE consensus on the definition of ’poor response’ to ovarian stimulation for in vitro fertilization: The Bologna criteria. Hum. Reprod..

[13] Esteves S.C., Roque M., Bedoschi G.M., Conforti A., Humaidan P., Alviggi C. (2018). Defining Low Prognosis Patients Undergoing Assisted Reproductive Technology: POSEIDON Criteria—The Why. Front. Endocrinol..

[14] Esteves S.C., Alviggi C., Humaidan P., Fischer R., Andersen C.Y., Conforti A., Bühler K., Sunkara S.K., Polyzos N.P., Galliano D. (2019). The POSEIDON Criteria and Its Measure of Success through the Eyes of Clinicians and Embryologists. Front. Endocrinol..

[15] Abrahams E. (2008). Right drug—right patient—right time: Personalized Medicine Coalition. Clin. Transl. Sci..

[16] Lemire F. (2013). The right drug for the right patient: Caring for our patients while minimizing prescription drug misuse. Can. Fam. Physician.

[17] Morón F.J., Ruiz A. (2010). Pharmacogenetics of controlled ovarian hyperstimulation: Time to corroborate the clinical utility of FSH receptor genetic markers. Pharmacogenomics.

[18] Nenonen H., Lindgren I.A., Prahl A.S., Trzybulska D., Kharraziha I., Hultén M., Giwercman Y.L., Henic E. (2019). The N680S variant in the follicle-stimulating hormone receptor gene identifies hyperresponders to controlled ovarian stimulation. Pharm. Genom..

[19] Achrekar S.K., Modi D.N., Desai S.K., Mangoli V.S., Mangoli R.V., Mahale S.D. (2009). Poor ovarian response to gonadotrophin stimulation is associated with FSH receptor polymorphism. Reprod. Biomed. Online.

[20] Riccetti L., De Pascali F., Gilioli L., Santi D., Brigante G., Simoni M., Casarini L. (2017). Genetics of gonadotropins and their receptors as markers of ovarian reserve and response in controlled ovarian stimulation. Best Pr. Res. Clin. Obstet. Gynaecol..

[21] Alviggi C., Conforti A., Santi D., Esteves S.C., Andersen C.Y., Humaidan P., Chiodini P., De Placido G., Simoni M. (2018). Clinical relevance of genetic variants of gonadotrophins and their receptors in controlled ovarian stimulation: A systematic review and meta-analysis. Hum. Reprod. Updat..

[22] Gerasimova T., Thanasoula M.N., Zattas D., Seli E., Sakkas D., Lalioti M.D. (2010). Identification and in vitro characterization of follicle stimulating hormone (FSH) receptor variants associated with abnormal ovarian response to FSH. J. Clin. Endocrinol. Metab..

[23] Lalioti M., Gerasimova T., Zattas D., Anastasakis D., Seli E., Sakkas D. (2010). A Deleted Form of FSH Receptor, Found in Women Undergoing Infertility Treatment, Impairs the Function of the Normal Receptor When Co-Expressed In Vitro. Biol. Reprod..

[24] Kuechler A., Hauffa B.P., Köninger A., Kleinau G., Albrecht B., Horsthemke B., Gromoll J. (2010). An unbalanced translocation unmasks a recessive mutation in the follicle-stimulating hormone receptor (FSHR) gene and causes FSH resistance. Eur. J. Hum. Genet..

[25] Beau I., Touraine P., Meduri G., Gougeon A., Desroches A., Matuchansky C., Milgrom E., Kuttenn F., Misrahi M. (1998). A novel phenotype related to partial loss of function mutations of the follicle stimulating hormone receptor. J. Clin. Investig..

[26] Ramadhan R.S. (2018). Molecular analysis of FSH receptor gene in Iraqi women with PCOS syndrome. Middle East Fertil. Soc. J..

[27] Čuš M., Vlaisavljević V., Repnik K., Potočnik U., Kovačič B. (2019). Could polymorphisms of some hormonal receptor genes, involved in folliculogenesis help in predicting patient response to controlled ovarian stimulation?. J. Assist. Reprod. Genet..

[28] De Castro F., Morón F.J., Montoro L., Galán J.J., Hernández D.P., Padilla E.S.-C., Ramírez-Lorca R., Real L.M., Ruiz A. (2004). Human controlled ovarian hyperstimulation outcome is a polygenic trait. Pharmacogenetics.

[29] Loutradis D., Patsoula E., Minas V., Koussidis G.A., Antsaklis A., Michalas S., Makrigiannakis A. (2006). FSH receptor gene polymorphisms have a role for different ovarian response to stimulation in patients entering IVF/ICSI-ET programs. J. Assist. Reprod. Genet..

[30] Livshyts G.B., Podlesnaja S., Kravchenko S., Sudoma I., Livshits L. (2008). A distribution of two SNPs in exon 10 of the FSHR gene among the women with a diminished ovarian reserve in Ukraine. J. Assist. Reprod. Genet..

[31] Mayorga M.P., Gromoll J., Behre H.M., Gassner C., Nieschlag E., Simoni M. (2000). Ovarian Response to Follicle-Stimulating Hormone (FSH) Stimulation Depends on the FSH Receptor Genotype*. J. Clin. Endocrinol. Metab..

[32] Behre H.M., Greb R.R., Mempel A., Sonntag B., Kiesel L., Kaltwasser P., Seliger E., Röpke F., Gromoll J., Nieschlag E. (2005). Significance of a common single nucleotide polymorphism in exon 10 of the follicle-stimulating hormone (FSH) receptor gene for the ovarian response to FSH: A pharmacogenetic approach to controlled ovarian hyperstimulation. Pharmacogenetics Genom..

[33] Yao Y., Ma C.-H., Tang H., Hu Y.-F. (2011). Influence of follicle-stimulating hormone receptor (FSHR) Ser680Asn polymorphism on ovarian function and in-vitro fertilization outcome: A meta-analysis. Mol. Genet. Metab..

[34] Altmäe S., Hovatta O., Stavreus-Evers A., Salumets A. (2011). Genetic predictors of controlled ovarian hyperstimulation: Where do we stand today?. Hum. Reprod. Updat..

[35] Lussiana C., Guani B., Mari C., Restagno G., Massobrio M., Revelli A. (2008). Mutations and polymorphisms of the FSH receptor (FSHR) gene: Clinical implications in female fecundity and molecular biology of FSHR protein and gene. Obstet. Gynecol. Surv..

[36] Ilgaz N.S., Aydos O.S.E., Karadag A., Taspinar M., Eryilmaz O.G., Sunguroglu A. (2015). Impact of follicle-stimulating hormone receptor variants in female infertility. J. Assist. Reprod. Genet..

[37] Desai S.S., Roy B.S., Mahale S.D. (2013). Mutations and polymorphisms in FSH receptor: Functional implications in human reproduction. Reproduction.

[38] Rizk B. (2009). Genetics of ovarian hyperstimulation syndrome. Reprod. Biomed. Online.

[39] Jun J.K., Yoon J.S., Ku S.-Y., Choi Y.M., Hwang K.R., Park S.Y., Lee G.H., Lee W.D., Kim S.H., Kim J.G. (2006). Follicle-stimulating hormone receptor gene polymorphism and ovarian responses to controlled ovarian hyperstimulation for IVF-ET. J. Hum. Genet..

[40] Tranchant T., Durand G., Gauthier C., Crépieux P., Ulloa-Aguirre A., Royère D., Reiter E. (2011). Preferential β-arrestin signalling at low receptor density revealed by functional characterization of the human FSH receptor A189 V mutation☆. Mol. Cell. Endocrinol..

[41] Bogerd J. (2007). Ligand-selective determinants in gonadotropin receptors. Mol. Cell. Endocrinol..

[42] Braun T., Schofield P.R., Sprengel R. (1991). Amino-terminal leucine-rich repeats in gonadotropin receptors determine hormone selectivity. EMBO J..

[43] Lazaros L., Hatzi E., Xita N., Takenaka A., Sofikitis N., Zikopoulos K., Georgiou I. (2013). Influence of FSHR diplotypes on ovarian response to standard gonadotropin stimulation for IVF/ICSI. J. Reprod. Med..

[44] Valkenburg O. (Olivier), Uitterlinden A., Piersma D., Hofman A., Themmen A., De Jong F., Fauser B., Laven J. (2009). Genetic polymorphisms of GnRH and gonadotrophic hormone receptors affect the phenotype of polycystic ovary syndrome. Hum. Reprod..

[45] Casarini L., Santi D., Marino M. (2015). Impact of gene polymorphisms of gonadotropins and their receptors on human reproductive success. Reproduction.

[46] He W.-B., Du J., Yang X.-W., Li W., Tang W.-L., Dai C., Chen Y.-Z., Zhang Y.-X., Lu G.-X., Lin G. (2019). Novel inactivating mutations in the FSH receptor cause premature ovarian insufficiency with resistant ovary syndrome. Reprod. Biomed. Online.

[47] Zariñán T., Mayorga J., Jardón-Valadez E., Gutiérrez-Sagal R., Maravillas-Montero J.L., Mejía-Domínguez N.R., Martínez-Luis I., Yacini-Torres O.G., Cravioto M.-D.-C., Reiter E. (2020). A Novel Mutation in the FSH Receptor (I423T) Affecting Receptor Activation and Leading to Primary Ovarian Failure. J. Clin. Endocrinol. Metab..

[48] Patel B., Parets S., Akana M., Kellogg G., Jansen M., Chang C., Cai Y., Fox R., Niknazar M., Shraga R. (2018). Comprehensive genetic testing for female and male infertility using next-generation sequencing. J. Assist. Reprod. Genet..

[49] Friemel C., Ammerpohl O., Gutwein J., Schmutzler A.G., Caliebe A., Kautza M., Von Otte S., Siebert R., Bens S. (2014). Array-based DNA methylation profiling in male infertility reveals allele-specific DNA methylation in PIWIL1 and PIWIL2. Fertil. Steril..

[50] Flugent. https://www.fulgentgenetics.com/Infertility-Female.

[51] Centogene. https://www.centogene.com/diagnostics/infertility-testing.html.

[52] CGCgenetics. https://www.cgcgenetics.com/en/by-test-a-z/5099.

[53] França M.M., Lerário A.M., Funari M.F., Nishi M.Y., Narcizo A.M., De Mello M.P., Guerra-Junior G., Maciel-Guerra A.T., De Mendonça B.B. (2017). A Novel Homozygous Missense FSHR Variant Associated with Hypergonadotropic Hypogonadism in Two Siblings from a Brazilian Family. Sex. Dev..

[54] Liu H., Xu X., Han T., Yan L., Cheng L., Qin Y., Liu W., Zhao S., Chen Z.-J. (2017). A novel homozygous mutation in the FSHR gene is causative for primary ovarian insufficiency. Fertil. Steril..

[55] Liu H., Guo T., Gong Z., Yu Y., Zhang Y., Zhao S., Qin Y. (2019). Novel FSHR mutations in Han Chinese women with sporadic premature ovarian insufficiency. Mol. Cell. Endocrinol..

[56] Lorenzi D., Fernández C., Bilinski M., Fabbro M., Galain M., Menazzi S., Miguens M., Perassi P.N., Fulco M.F., Kopelman S. (2020). First custom next-generation sequencing infertility panel in Latin America: Design and first results. JBRA Assist. Reprod..

[57] França M.M., Mendonca B.B. (2020). Genetics of Primary Ovarian Insufficiency in the Next-Generation Sequencing Era. J. Endocr. Soc..

[58] Reisberg S., Krebs K., Lepamets M., Kals M., Mägi R., Metsalu K., Lauschke V.M., Vilo J., Milani L. (2019). Translating genotype data of 44,000 biobank participants into clinical pharmacogenetic recommendations: Challenges and solutions. Genet. Med..

[59] Bramble M.S., Goldstein E.H., Lipson A., Ngun T., Eskin A., Gosschalk J.E., Roach L., Vashist N., Barseghyan H., Lee E. (2016). A novel follicle-stimulating hormone receptor mutation causing primary ovarian failure: A fertility application of whole exome sequencing. Hum. Reprod..

[60] Zhou Y., Fujikura K., Mkrtchian S., Lauschke V.M. (2018). Computational Methods for the Pharmacogenetic Interpretation of Next Generation Sequencing Data. Front. Pharmacol..

[61] Ay B.G.B., May J.V., Davis J.S., Dias J.A., Kumar T.R. (2018). In Vivo and In Vitro Impact of Carbohydrate Variation on Human Follicle-Stimulating Hormone Function. Front. Endocrinol..

[62] Davis D., Liu X., Segaloff D.L. (1995). Identification of the sites of N-linked glycosylation on the follicle-stimulating hormone (FSH) receptor and assessment of their role in FSH receptor function. Mol. Endocrinol..

[63] Sayers N., Hanyaloglu A.C. (2018). Intracellular Follicle-Stimulating Hormone Receptor Trafficking and Signaling. Front. Endocrinol..

[64] Landomiel F., Gallay N., Jégot G., Tranchant T., Durand G., Bourquard T., Crépieux P., Poupon A., Reiter E. (2014). Biased signalling in follicle stimulating hormone action. Mol. Cell. Endocrinol..

[65] Alviggi C., Conforti A., Cariati F., Alfano S., Huhtaniemi I., Santi D., De Placido G., Humaidan P. Impact of polymorphisms of gonadotropins and their receptors on controlled ovarian stimulation: A prospective observational study. Proceedings of the 32nd Annual Meeting of ESHRE.

[66] Badawy A., Wageah A., El Gharib M., Osman E.E. (2011). Prediction and Diagnosis of Poor Ovarian Response: The Dilemma. J. Reprod. Infertil..

[67] Loutradis D., Drakakis P., Vomvolaki E., Antsaklis A. (2007). Different ovarian stimulation protocols for women with diminished ovarian reserve. J. Assist. Reprod. Genet..

[68] Conforti A., Esteves S.C., Cimadomo D., Vaiarelli A., Di Rella F., Ubaldi F.M., Zullo F., De Placido G., Alviggi C. (2019). Management of Women With an Unexpected Low Ovarian Response to Gonadotropin. Front. Endocrinol..

[69] García-Jiménez G., Zariñán T., Rodríguez-Valentín R., Mejía-Domínguez N.R., Gutiérrez-Sagal R., Hernández-Montes G., Tovar A.R., Arechavaleta-Velasco F., Canto P., Granados J. (2018). Frequency of the T307A, N680S, and -29G>A single-nucleotide polymorphisms in the follicle-stimulating hormone receptor in Mexican subjects of Hispanic ancestry. Reprod. Biol. Endocrinol..

[70] Paschalidou C., Anagnostou E., Mavrogianni D., Raouasnte R., Klimis N., Drakakis P., Loutradis D. (2020). The effects of follicle-stimulating hormone receptor (FSHR) -29 and Ser680Asn polymorphisms in IVF/ICSI. Horm. Mol. Biol. Clin. Investig..

[71] Ammar R., Paton T.A., Torti D., Shlien A., Bader G.D. (2015). Long read nanopore sequencing for detection of HLA and CYP2D6 variants and haplotypes. F1000Research.

[72] Conforti A., Vaiarelli A., Cimadomo D., Bagnulo F., Peluso S., Carbone L., Di Rella F., De Placido G., Ubaldi F.M., Huhtaniemi I. (2019). Pharmacogenetics of FSH Action in the Female. Front. Endocrinol..

